# Assessment of phytotoxicity of ZnO NPs on a medicinal plant, *Fagopyrum esculentum*

**DOI:** 10.1007/s11356-012-1069-8

**Published:** 2012-07-20

**Authors:** Sooyeon Lee, Sunghyun Kim, Saeyeon Kim, Insook Lee

**Affiliations:** 1 Division of EcoScience, Ewha Womans University, 52, Ewhayeodae-gil, Seodaemun-gu, Seoul, 120-750 South Korea; 2 School of Civil and Environmental Engineering, Yonsei University, Seoul, South Korea

**Keywords:** Buckwheat (*Fagopyrum esculentum*), ZnO nanoparticles, Phytotoxic indicator, Antioxidative enzyme

## Abstract

*Fagopyrum esculentum* commonly named as buckwheat plant is pseudocereal food crops and healthy herbs but is not known as a bioindicator of environmental condition. In the present study, the effects of ZnO nanoparticles (NPs) and microparticles (MPs) on plant growth, bioaccumulation, and antioxidative enzyme activity in buckwheat were estimated under hydroponic culture. The significant biomass reduction at concentrations of 10–2,000 mg/L was 7.7–26.4 % for the ZnO NP and 11.4–23.5 % for the ZnO MP treatment, (*p* < 0.05). ZnO NPs were observed in root cells and root cell surface by scanning electron microscopy and transmission electron microscopy analysis. Zn bioaccumulation in plant increased with increasing treatment concentrations. The upward translocation (translocation factor <0.2) of Zn in plant was higher with the ZnO NP treatment than that with the ZnO MP treatment. Additionally, reactive oxygen species generation by ZnO NPs was estimated as the reduced glutathione level and catalase activity, which would be a predictive biomarker of nanotoxicity. The results are the first study to evaluate the phytotoxicity of ZnO NPs to medicinal plant. *F*. *esculentum* can be as a good indicator of plant species in NP-polluted environment.

**Electronic supplementary material:**

The online version of this article (doi:10.1007/s11356-012-1069-8) contains supplementary material, which is available to authorized users.

## Introduction

*Fagopyrum esculentum* is commonly named as a buckwheat plant in the dicot family Polygonaceae: the Eurasian genus *Fagopyrum* (Quinet et al. 2004). Buckwheat is sometimes used as a green manure, as a medical uses for cholesterol capture, or as a pollen and nectar source for biological control (Tomotake et al. 2001; Berndt et al. 2002). In addition, the seed of *F*. *esculentum* has promising pharmacological efficacy and rich in mineral, aromatic compound, and antioxidants as tannins and rutin (Bonafaccia et al. 2003; Kreft et al. 1999). Generally, native crops and cultivated plants are used for bioindicator or biomonitor of pollution damage (Weinstein et al. 1990). Because, they are easy to grow and adaptable to environmental stress and can be used for assessment of environmental conditions in different habitats. Moreover, plant-based assays applied for toxicity evaluation in the fields would reduce animal sacrifices and testing costs (Grant 1994). Among plant species, buckwheat is known as metal hyperaccumulator and to be translocated well to shoots from roots (Tamura et al. 2005). However, the uptake, accumulation, and translocation of engineered nanoparticles (ENPs) by buckwheat are not investigated.

Nanoscience is becoming a major field of study and rapidly increasing number of ENPs from cosmetics to medicine and agriculture can be accidentally or incidentally released (Service 2008; Colvin 2003). Due to having considerable impact on health and the environment, public concern of such nanoparticles (NPs) also has stimulated research on nanotoxicology with a focus on mammalian cytotoxicity and impacts on animals and bacteria (Roco 2003). Even though plants serve as an important potential source of NP transport, they provide potential routes for bioaccumulation in the food chain (Zhu et al. 2008). Several recent studies have evaluated ENP phytotoxicity as well as their ecotoxicity (Barrena et al. 2009; Guangke et al. 2011; Lee et al. 2008). The effect of ENPs on different plant species varies greatly, and both positive and negative effects have been reported. Interestingly, NPs cause both positive (Khodakovskaya et al. 2009; Lu et al. 2002) and negative effects (Lee et al. 2010; Lin and Xing 2007; Yang and Watts 2005) on root elongation, depending on the plant species (corn, cucumber, soybean, cabbage, carrot, and tomato).

Among metal oxide NPs, ZnO NPs are widely used in cosmetic and skin care products such as in sunscreens, toothpastes, shampoos and soaps, anticancer medicines, and photocatalyst pigments. Other studies that have examined the uptake of ZnO NPs by ryegrass (*Lolium perenne*) reported no upward translocation of ZnO NPs from root to shoots. ZnO NPs primarily adhere to the root surface and are observed in the apoplast and protoplast spaces in root endodermis (Lin and Xing 2007). In addition, metal oxide NPs promote reactive oxygen species (ROS) generation, which is a predictive chemical marker of nanotoxicity and an indicator for evaluating ENP phytotoxicity (Adams et al. 2006; Choi and Hu 2008). Oxidative stress occurs when ROS disturb the balance between oxidative pressure and antioxidant defense (Singh et al. 2010; Gajewaska and Sklodowska 2007). To cope with ROS, plant cells possess an antioxidant defense capacity consisting of both enzymatic and nonenzymatic antioxidants such as catalase (CAT), glutathione reductase, glutathione-*S*-transferase, and peroxidase (Klaper et al. 2009). Oxidative stress is a state of redox disequilibrium during ROS production. The reduced glutathione (GSH) to glutathione disulfide (GSSG) ratio is a functional sensor of oxidative stress expression (Akerboom and Sies 1981). Thus, it is important for estimating whether medicinal plants are protected from nanotoxic materials or not. Therefore, studies that examine the effect of ZnO NP on growth and oxidative stress in buckwheat of medicinal plants must be conducted. In the present study, we compared the phytotoxic effects of ZnO particles on buck wheat (*F. esculentum*) seedlings. The aim of this study was to assess the phytotoxicity of ZnO NP on a medicinal plant by investigating the effects of biomass, bioaccumulation, and antioxidative enzyme activity.

## Materials and methods

### Experimental design

**Fig. 1:**
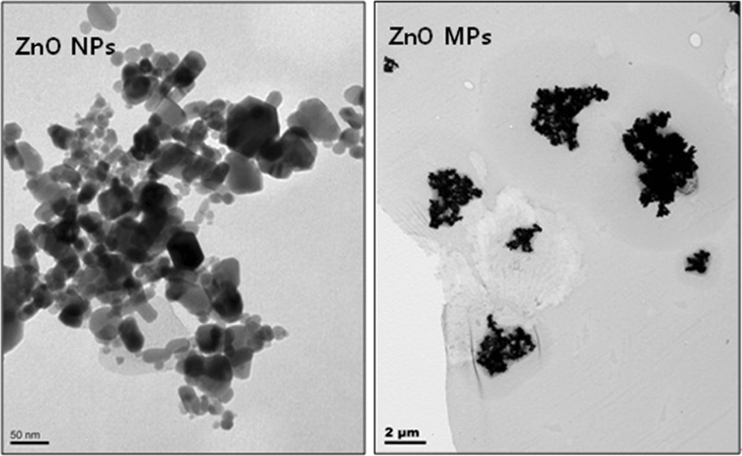
TEM images of ZnO NPs and MPs (500 mg/L) in nutrition solution for particle size and morphology determination. *Bars* show the scale in nanometers

The ZnO NPs and microparticles (MPs) were purchased from Sigma Aldrich Chemical Co. (St. Louis, MO, USA) and stored according to the vendor's instructions. Nanoparticles size and morphology were characterized by transmission electron microscopy (TEM; LIBRA 120, Carl Zeiss, Germany) in 1/2 strength Hoagland solution at concentration 500 mg/L (Fig. 1). In order to provide more reliable data on phytotoxicity of NPs, a morphology characterization of the NPs in solution (colloidal stability, surface potential, and diameter) is required (Sayes and Warheit 2009). In nutrition solution state, individual of ZnO NPs are rod and nonspherical in shape, and ZnO MPs are also nonspherical particles with little extent of aggregation than that of NPs. The particle size of ZnO NPs was 44.46 ± 4.84 nm and ZnO MPs's particle size was 2 ~ 5 μm, which were almost the same as that (ZnO NPs, <50 nm; ZnO MPs, <5 μm) from producer. ZnO NPs purity was >97 % and the specific surface area was >10.8 m^2^ g^−1^. ZnO MPs had a purity of 99 % and a specific surface area of 3–4 m^2^ g^−1^. ZnO particles were prepared in 1/2 strength Hoagland solution (Fossati et al. 1980) at treatment concentrations of 0, 1, 5, 100, 1,000, and 2,000 mg/L separately to disperse the particles for hydroponic culture. For protection of aggregation of ZnO NP, suspensions were homogenized by ultrasonication (100 W, 40 kHz) for 30 min and then filtered with 0.45 μm nylon membrane filter (Whatman International, Maidstone, Kent, UK). The concentrations of ion in the filtrates were measured via atomic absorption spectroscopy (Analysis 100, Perkin-Elmer Inc., Waltham, MA, USA). The pH of all stock suspensions was approximately 6.5.

The plant species used was buckwheat (*F. esculentum*). This species was selected as a model plant system due to its wide distribution and common use in phytotoxicity studies (EPA 1996). Seeds of *F. esculentum* were purchased from local stores and sown in a pot containing natural soil in a greenhouse maintained at 25 °C for 2 weeks. The plants were transferred to three 50 mL Falcon tubes with four seedlings in 40 mL of 1/2 strength Hoagland solution for hydroponic culture. The hydroponic seedlings in Hoagland solution were treated with nano- and micro-sized ZnO particles at concentrations of 0–2,000 mg/L.

In addition, root tissues were further observed by scanning electron (SEM) and TEM to determine if ZnO NPs entered the plant cells. TEM samples were prepared following standard procedures. *F. esculentum* root samples were prefixed in 2 % (*v*/*v*) glutaraldehyde for 2 h, washed in 0.1 M phosphate buffer at pH 7.2, post-fixed in 1 % osmium tetraoxide for 2 h, dehydrated in acetone, and infiltrated and embedded in epoxy resin. After polymerization, root tip samples were observed by SEM (Aurigia, Carl Zeiss, Jena, Germany). Samples were observed by TEM (Libra 120, Carl Zeiss) after being sectioned (Ultra microtome: MT-X, RMC, Tucson, AZ, USA).

### Phytotoxicity tests

#### Biomass and Zn concentration determination

After 5 days, the plants were harvested, washed with deionized water, and the water was removed. The fresh weight of the plant material was determined using a balance three times in succession. The length of the shoots and roots was measured three times.

To investigate the transfer of ZnO NPs and MPs into plants, Zn accumulation of *F. esculentum* treated with ZnO NPs and MPs and control (No ZnO) was analyzed. After the shoots and roots were dried at 70 °C for 48 h, the samples were digested using a HNO_3_ microwave digestion system (MDS-2000, CEM Inc., Matthews, NC, USA) with tetrafluormethaxil vessels, and the supernatants were subsequently filtered through 0.45 μm PVDF syringe filters (Whatman International, Maidstone, Kent, UK). The system was calibrated using certified reference materials (SRM 1573a: tomato leaves) obtained from the National Institute for Environmental Studies of Japan. Recovered Zn was obtained with a yield of 94.4 %.

#### Antioxidative enzyme activity assays

Fresh *F. esculentum* plants (0.1 g) exposed to ZnO particles and control (No ZnO) were homogenized in 1.5 mL of ice-cold extraction buffer containing 100 mM potassium phosphate buffer (pH 7.0) containing 5 mM EDTA. The homogenates were centrifuged at 10,000× *g* for 30 min, and the supernatant was stored in separate aliquots at −80 °C prior to analysis.

A reduced GSH assay (CS0260, Sigma Aldrich Chemical Co. St. Louis, MO, USA) was used to determine the level of total GSH (GSSG + GSH) in the cell. In this kinetic assay, the catalytic amount (in nanomoles) of GSH resulting from continuous reduction of 5,5′-dithiobis (2-nitrobenzoic acid) to thionitrobenzoic acid (TNB) is measured, and the GSSG formed is recycled by glutathione reductase and NADPH (Akerboom and Sies 1981). The yellow product, TNB, is measured at 412 nm using a spectrophotometer.

CAT activity was determined using a colorimetric method that measures hydrogen peroxide consumption at 520 nm (Fossati et al. 1980). CAT activity (CAT100, Sigma Aldrich Chemical Co. St. Louis, MO, USA) was assayed for 5 min in a CAT reaction solution composed of 50 mM potassium phosphate buffer (pH 7.0), 200 mM H_2_O_2_, and 50 μL of crude extract. One unit of catalase will decompose 1.0 μmol of hydrogen peroxide to oxygen and water per minute at pH 7.0 at 25 °C at a substrate concentration of 50 mM hydrogen peroxide.

### Statistical analysis

Each concentration of the three treatments was conducted in triplicate (three tubes with four seedlings each). Turkey's post hoc test and an analysis of one-way ANOVA were used to test for differences using SPSS version 12.0 software (SPSS Inc., Chicago, IL, USA).

## Results and discussion

**Fig. 2:**
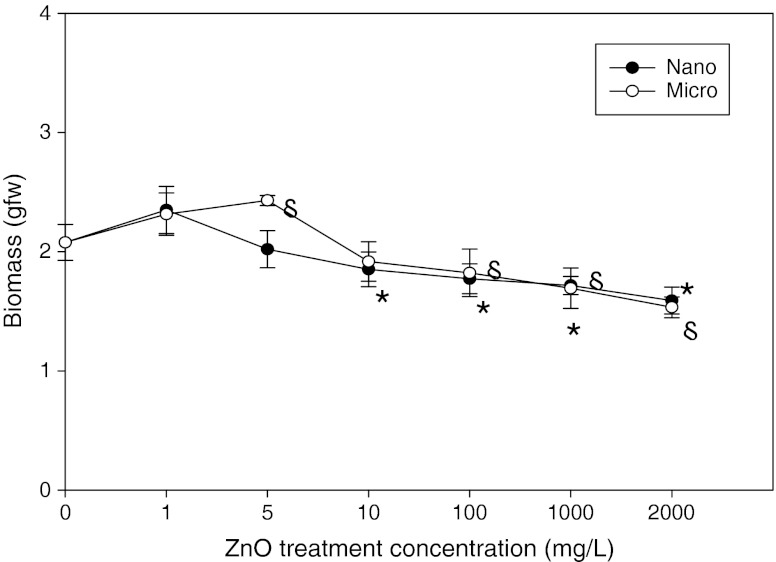
Biomass of *F. esculentum* exposed to different concentrations of ZnO particles. One set included four seedlings and experiments were performed in triplicate. Data are the mean and SD, *P < 0.05, ZnO NPs treatment vs. control, §*P* < 0.05, ZnO MPs treatment vs. control (significantly different)

Plants expose huge interfaces to the air and soil environment. Thus, persistent NPs with crop plants can enter the human food chain. The accurate assessment of the toxic effect of NPs in plant should be conducted. In this experiment, biomass and translocation factor (TF) against size and concentration of ZnO NPs and ZnO MP were measured. And the antioxidant enzyme activity, which indicated the production of ROS responding to ZnO NPs, were estimated. Our results showed that biomass and TF were decrease in response to ZnO high concentration. The seedling growth (biomass) of *F. esculentum* decreased with an increase in the ZnO NP and MP concentrations (Figs. 2 and 5). This result was in agreement with a study by Lin and Xing (2007), who reported no significant root inhibition in rape and ryegrass when the treatment concentration was <10 mg/L and for radish when the treatment concentration was <20 mg/L. A positive effect of multi-walled carbon nanotubes is observed at concentrations 10–40 mg/L on seed germination and tomato leaf growth (Khodakovskaya et al. 2009). Interestingly, the biomass of *F. esculentum* treated with 1 mg/L ZnO NPs (2.35 g ± 0.2) and 1 and 5 mg/L ZnO MPs (2.32 g ± 0.2 and 2.43 g ± 0.1, respectively) was rather higher than that of the control (2.08 g ± 0.2), suggesting that no decrease in biomass occurred at low concentrations due to the essential elemental nutrition in the ZnO particles. The significant biomass reduction in *F. esculentum* at concentrations of 10–2,000 mg/L was 7.7–26.4 % for the ZnO NP and 11.4–23.5 % for the ZnO MP treatment (*p* < 0.05). The morphology of the samples after the ZnO NP and MP treatments also was different from that of the control (data not shown). In particular, the roots were shortened and damaged in plants exposed to the 1,000 and 2,000 mg/L ZnO NP treatment concentrations. These results are in agreement with a report by Lin and Xing (2008), who found morphological changes (root cap deformity) in ryegrass root tips exposed to 1,000 mg/L ZnO NP. Phytotoxic phenotypes in seedlings caused by ENPs include stunted growth, reduced biomass, and root cap deformities (Barrena et al. 2009). In addition, the dissolution of ZnO NPs was estimated whether phytotoxic effects are due to ZnO NPs itself or Zn^2+^ ion dissolved from NPs surfaces. ZnO NP and MP suspensions released 0.58 to 3.85 μg/mL of concentration Zn^2+^-free ion in all treatments. This range supported the result that the bacterial growth slightly inhibition by free ion toxicity is negligible under the experimental concentration (Baek and An 2011). Therefore, it suggests that ion-inducible toxicity was not significant in present study.

**Fig. 3:**
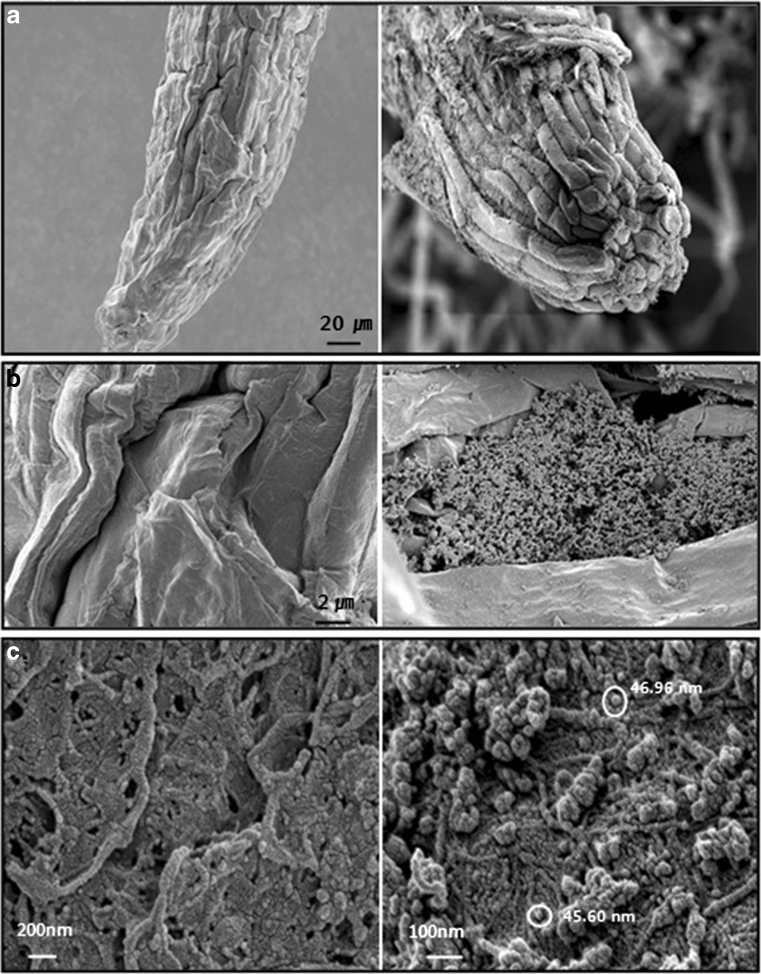
SEM analysis of *F. esculentum* root surface under control (*left*) and treatment (*right*) with ZnO NPs (1,000 mg/L) at a magnification of × 1,000 (**a**), ×5,000 (**b**), and × 150,000 (**c**)

SEM images of the root surface were acquired at a magnification of ×1,000 (a), ×5,000 (b), and ×150,000 (c) to investigate the phytotoxicity of ZnO NPs (Fig. 3). The surfaces of the root epidermal cells treated with a high dose (1,000 mg/L) NPs were more different in morphology than that of the control. The root surface of the control was free of particles. However, the NPs had adhered onto the root cap surface, which may result in root growth inhibition directly. The particles filled the epidermal crypt on the root surface, which supported the results observed on SEM images of the root surface of ryegrass after treatment with ZnO NPs (Lin and Xing 2008). The NPs aggregates would deposit quickly without stirring the solution during observation and this aggregates may influence their translocation and morphology by changing the plant cell's subcellular organization (Lin and Xing 2008; Corredor et al. 2009; Aubert et al. 2012). ZnO NPs in nutrient solution were more aggregated than those in deionized water. This result was supported by the fact that the aggregation of NPs in aqueous phase may influence their phytotoxicity by the report of Kim et al. (2012).

**Fig. 4:**
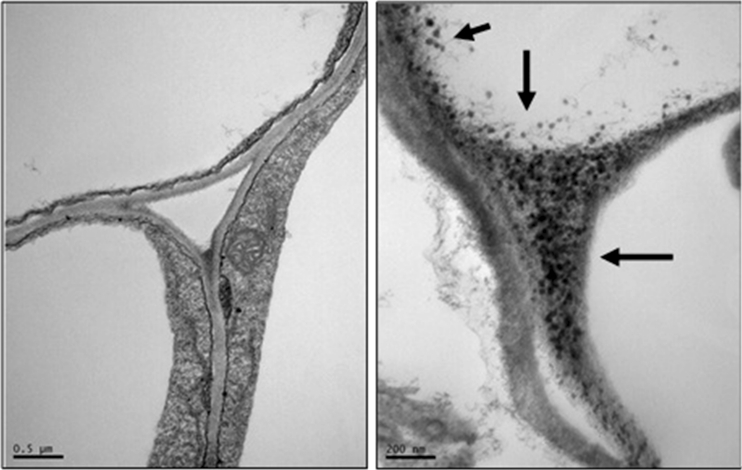
TEM analysis of *F. esculentum* root surface under control (*left*) and treatment (*right*) with ZnO NPs (1,000 mg/L)

A TEM analysis was performed to evaluate the immobilization and aggregation of NPs in root tissue (Fig. 4). At right panel of treatment with ZnO NPs (1,000 mg/L), the dark dots (solid arrow) are clearly visible in the cytoplasm. Variable-type NPs' presence was identified in the dark dots as shown in the higher magnification TEM images (Supporting Information Fig. 1). It suggests that NPs can pass the cell membrane and formed agglomerates, with other cellular materials within the cells. In a word, this amount of individual NPs may have formed secondary-sized NPs (aggregates) in the cell. This could be more a toxic effect of the ZnO NPs than that of control.

**Fig. 5:**
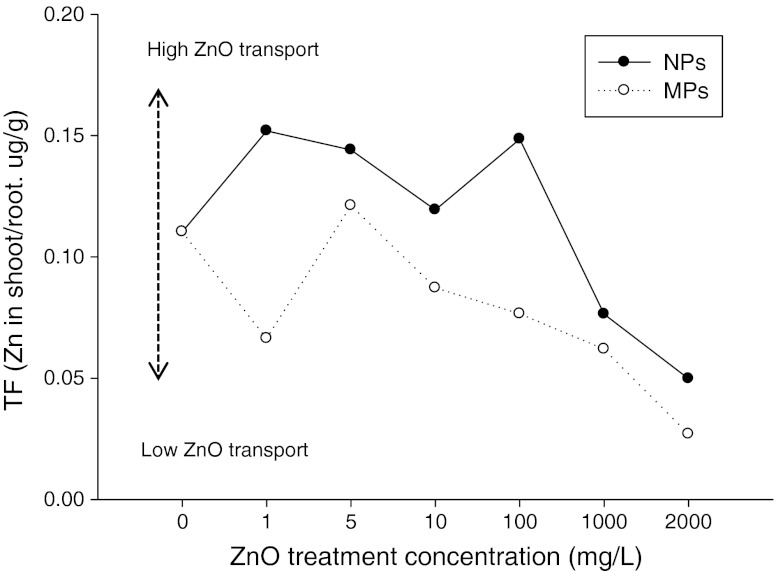
Translocation factor (*TF* ratio Zn content in shoot to Zn content in root) of *F. esculentum* exposed to the control and six concentrations of ZnO NPs and MPs

Zn accumulation in *F. esculentum* increased with increasing treatment concentration. Zn content in the root was slightly higher in the ZnO MPs treatment than those in the NP treatment. Uptake into plants was measured by calculating the TF, which is defined as the ratio of Zn concentration in the shoots to that in roots (Tilney et al. 1991). However, TF of ZnO NPs was higher (1.19–2.29 times) than that of ZnO MPs at all treatment concentrations. The upward translocation (TF <0.2) of Zn in buckwheat was higher with the ZnO NP treatment than that with the ZnO MP treatment (Fig. 5). However, the upward translocation of Zn NPs remained very low (TF <0.02) in the study by Lin and Xing (2008). This may be the case because buckwheat contained more accumulated species than ryegrass even though aggregation of ZnO NPs was observed in both samples. Additionally, the Zn TF decreased with increasing concentration of ZnO NPs and MPs. The difference in translocation of NPs of different sizes may affect their toxicity and availability in plant tissues. Due to their small size and aggregation in the aqueous phase, the bioavailability and translocation pattern of NPs are correlated with toxicity (Kim et al. 2009; Lin and Xing 2008; Ma et al. 2010; Rico et al. 2011). Therefore, ZnO particle accumulation in buckwheat, which can be used as a model crop plant to assess bioaccumulation in ecosystems, may be important for understanding the different translocation patterns of other crop species.

Antioxidative enzyme activity was measured to investigate the phytotoxicity of ZnO NPs in buckwheat (Table 1). Because, the elevated ROS level as first defense mechanism was claimed to be of major importance in the toxicological profile (Xia et al. 2006; Gajewaska and Sklodowska 2007). Reduce GSH is the major free thiol in most living cells and is involved in many biological processes detoxification of xenobiotic, removal of H_2_O_2_. Thus, intracellular GSH status can be a sensitive indicator of the overall health of a cell and of its ability to resist toxic challenge (Akerboom and Sies 1981). In our study, the increase in GSH in buckwheat seedling treated with NPs was notably increased than that of the control (*P* < 0.05). At the high NP doses (1,000 or 2,000 mg/L), the amount of GSH was lower (49.6 ± 2.6 μmol/mg protein) than that of low NP doses (1 ~ 100 mg/L). This result suggests that a high dose of ZnO NPs might have uncontrollably stimulated the antioxidant defense system (Xia et al. 2006). This phenomenon was also observed for CAT activity after treatments of 1,000 and 2,000 mg/L of ZnO NPs. CAT enzyme activity was increased at all treatment concentrations. CAT is also an important enzyme in antioxidant defense systems by converting free radicals H_2_O_2_ to water and oxygen (H_2_O + O_2_). Thus, it provides protection against oxidative damage to the cell (Bai et al. 1999). In this study, there was significantly increase in CAT level at all test concentration compared to control. However, CAT activity seemed to be a saturated pattern at the high dose (1,000 and 2,000 mg/L) NPs. This phenomenon resulted from high dose of NPs that produced too much ROS, which exceeded the scavenging capacity of CAT, inhibited its activity, and produced oxidative damage (Xia et al. 2006; Kim et al. 2009). Therefore, we suggested that antioxidant enzyme is a predictive biomarker for oxidative stress potential in buckwheat exposure to ZnO NPs.

**Table 1:** Antioxidative enzyme activity of 20-day seedlings of *F. esculentum* exposed to the control and six concentrations of ZnO NPs for 5 days

ZnO NPs treatment (mg/L)	GSH concentration (μmol/mg protein)	Catalse activity (unit/mg protein)
Control	31.3 ± 9.7 a	99.5 ± 8.1 a
1	31.2 ± 7.1 a	140.1 ± 10.0 b
5	45.3 ± 5.4 b	109.3 ± 4.2 a
10	53.0 ± 8.0 c	136.7 ± 23.0 b
100	61.6 ± 7.6 c	152.8 ± 28.1 b
1,000	61.6 ± 3.8 c	109.2 ± 12.7 a
2,000	49.6 ± 2.6 b	108.0 ± 3.5 a

GSH level and catalase activity are shown as the mean and SD. The different small letters represent statistical significant differences (*P* < 0.05) from the Turkey's test

## Conclusion

This study was aimed at assessing the phytotoxicity of ZnO NP on a medicinal plant by investigating the effects of biomass, bioaccumulation, and antioxidative enzyme activity. The biomass of buckwheat seedlings was more significantly reduced in response to ZnO NPs than to MPs at concentrations of 10–2,000 mg/L. SEM and TEM analyses revealed that the surfaces of root cells exposed to high dose (1,000 mg m/L) NPs were more damaged by NP aggregates than those in the control. In addition, Zn uptake by *F. esculentum* roots was higher in response to ZnO MPs treatment than to NPs treatment. However, the upward TFs of Zn from roots to shoots were higher for the ZnO NP treatment than for the ZnO MP treatment. ROS enzyme activity (CAT) and antioxidants (GSH) increased, which are dependent on particle concentration. High dose ZnO NPs may have uncontrollably induced the ROS defense system. In this work, the results demonstrated that the first study evaluated the phytotoxicity of NPs to medicinal plant, *F*. *esculentum*, as a good indicator plant species in NP-polluted environment.

## Electronic supplementary material

Below is the link to the electronic supplementary material.

Supporting Information Fig. 1 TEM images for ZnO NPs treatments shows a significant different from control for statistical analysis. NPs (solid arrow) are clearly visible in the cytoplasm. (DOCX 86 kb)
