# Supplementary material for: Assessment of phytotoxicity of ZnO NPs on a medicinal plant, Fagopyrum esculentum
Source: Environ Sci Pollut Res Int. 2012 Jul 20;20(2):848–54. doi: 10.1007/s11356-012-1069-8 (PMC3555345; doi:10.1007/s11356-012-1069-8)
Supplement: Supplementary file 1 — TEM images for ZnO NPs treatments shows a significant different from control for statistical analysis. NPs (solid arrow) are clearly visible in the cytoplasm. (DOCX 86 kb) [file 11356_2012_1069_MOESM1_ESM.docx]

**Supporting Information**

**Fig. 1** TEM images for ZnO NPs treatments shows a significant different from control for statistical analysis. NPs (solid arrow) are clearly visible in the cytoplasm

**
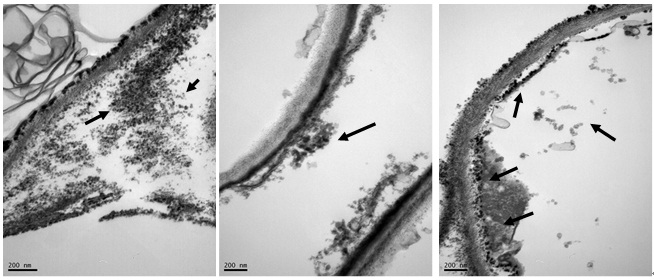
**
